# A question of fate

**DOI:** 10.1371/journal.pbio.2002329

**Published:** 2017-05-15

**Authors:** Mirjana Maletic-Savatic

**Affiliations:** Baylor College of Medicine, Jan and Dan Duncan Neurological Research Institute at Texas Children's Hospital, Houston, Texas, United States of America

## Abstract

Ever since the discovery of neural stem cells in the mammalian brain, the possibility of brain tissue regeneration has captured the minds of scientists, clinicians, and the public. Neural stem cells have been envisioned as a source of donor cells for transplantation and vectors for the delivery of gene therapy. Over the past decade, many researchers have contributed to characterizing these cells and their lineages, providing the foundation for their utilization as therapeutic devices. In a new study, Azim and colleagues took a different approach: using pharmacogenomics to focus on neural stem cell lineage, they identified specific compounds that can direct neural stem cell fate toward a specific lineage in vivo, both in physiological and pathological conditions. Their work opens new avenues for treatment of neurodegenerative and demyelinating disorders.

The majority of cells of the nervous system (neurons, astrocytes, and oligodendrocytes) derive from neural stem cells (NSCs). Not only do NSCs line the neural tube during early mammalian development to give rise to the rest of the central nervous system [1], but they continue to generate new neurons throughout the lifespan within the 2 neurogenic niches: the subventricular zone (SVZ) and the subgranular zone (SGZ) of the dentate gyrus [2,3]. The discovery that the brain is actually capable of generating new neurons in adulthood [4] was initially met with vehement skepticism, but that skepticism has given way to hope that NSCs can be manipulated to replace cells lost to a wide variety of insults [5–9], much like the way other tissue-specific stem cells are being studied for clinical applications.

If adult NSCs and their progeny are to be used as a therapeutic modality for brain repair, certain challenges to manipulating the NSC population must be overcome before they can be employed reliably for therapy. First, the heterogeneity of NSCs and their immediate progeny, transient amplifying cells (TAPs), along with their small population number, has made it difficult to precisely define these cells [10]. Second, therapeutic reliability and safety require that we be able to control the fate of the transplanted cells, as the risk of uncontrolled growth or transformation into a different identity is a concern for all regenerative therapies. To achieve such control requires a much better understanding of the mechanisms that stimulate NSCs than we currently possess. Nevertheless, NSCs injected intravenously or directly into the brain of various animal models have been able to survive, migrate towards injury, and differentiate into neurons [11–15], proving that hopes of regenerative therapy are realistic.

At present, it is clear that the varying lifespans, lineage plasticity, and regenerative potential of the different cell types depend on their origin, age, and exposure to myriad internal and external stimuli [10]. In this issue of *PLOS Biology*, Azim and collaborators [16] bring us a step closer to clinically applicable stem-cell therapies by focusing on functional outcomes of NSCs during early postnatal periods: they examined NSC lineages in the SVZ microdomains (dorsal versus ventral/lateral) and identified small molecules that direct the fate of these cells toward neurogenic as opposed to oligodendrogenic lineages.

They accomplished this feat by taking advantage of the spatiotemporal specificity of NSCs during the early postnatal period: stem cells in the ventral/lateral SVZ give rise to interneurons of the olfactory bulb and cortical areas [17,18], whereas NSCs in the dorsal SVZ produce glutamatergic neurons and oligodendrocytes. Azim et al. [16] performed transcriptome analysis of NSCs and TAPs from the discrete SVZ microdomains at several early postnatal time points. They then compared the profiles to identify pathways unique to each set of cells, confirming significant regional specificity in the different populations as well as their propensity toward a certain lineage (neuronal versus oligodendroglial). With these microarray datasets in hand, the authors set out to identify small molecule drug-like "perturbagens" that reproduce the observed regional and/or lineage-specific transcriptional changes. They took advantage of the pharmacogenomics approach (Box 1) and 2 public databases. The first was the Connectivity Map (CMAP) database of drug-associated transcriptional profiles in human cell lines [19]. The motivating idea for CMAP is that if a disease state manifests in a well-defined transcriptional response, then a drug that has the opposite effect on the transcriptome might be of therapeutic value. The authors queried CMAP for drugs with transcriptional profiles similar to those determining cell fate. The authors then used a second platform, the Searchable Platform Independent Expression Database (SPIED), to interrogate CMAP with gene-based expression profiles. SPIED’s greatly extended set of expression data allowed for the cell fate determining profiles to be put in a wider context of publicly available expression data [20].

**Box 1. What is pharmacogenomics?**

The realization that differences in individual makeup can influence the response to a given compound has been with us since Pythagoras, who recognized that ingestion of fava beans could lead to jaundice in some individuals. After 2,500 years, this astute observation was confirmed in people with deficiency of glucose-6-phosphate dehydrogenase and called favism. With the subsequent development of the field of genetics, a new discipline, pharmacogenetics—the study of how variations in one gene can influence an individual’s response to a single drug—was conceived [23]. More recently, systems biology approaches and ‘omics sciences have led to the current field of pharmacogenomics, which studies how all of the genes (the genome) can influence responses to drugs [24].

Pharmacogenomics burgeoned after the publication of the first Connectivity Map (CMAP) database, consisting of the gene expression profiles of 5 cancer cell lines treated by 1,309 small molecules (“perturbagens”) along with pattern-matching algorithms to detect similarities among the signatures [19]. This was the first time that drug effects could be correlated with gene expression patterns on such a large level. Since then, a flurry of studies has led to multiple applications of pharmacogenomics and the expansion of the published expression studies in Gene Expression data Mining Toward Relevant Network Discovery (GEM-TREND) [25], Profil-Chaser [26], the Searchable Platform Independent Expression Database (SPIED) [20], and Library of Integrated Network-based Cellular Signatures, (LINCS) which covers multiple cellular contexts and time points across multiple compounds (www.lincsproject.org). Pharmacogenomics is proving particularly useful in several areas. First, genomic information can *facilitate the discovery of new drug targets* by uniting early-phase data and guided selection of drug targets, which in turn should lead to lower drug failure rates caused by lack of efficacy. As a matter of fact, genomic data may actually help identify the *basis for lack of efficacy or occurrence of adverse reactions*. Second, pharmacogenomics can provide supporting evidence to *link a drug target to clinically relevant outcomes*. Such associations can provide meaningful insights into the value of a drug target that go beyond that of the association with a biomarker. Finally, pharmacogenomics can *improve clinical trial design* by enabling selection of patients more likely to experience benefit from the given drug or less likely to experience adverse events, such as for Dal-GenE trial (http://www.hra.nhs.uk/news/research-summaries/the-dal-gene-trial/). Indeed, a recent study reported that drug targets that have made it further along the drug development pipeline are more likely to have mechanistic support from genetic evidence and were predicted to reach regulatory approval twice as often as those without [27].

In early 2015, President Obama announced a research initiative to accelerate progress toward personalized and precision medicine (https://obamawhitehouse.archives.gov/precision-medicine), built on the foundation of the human genome sequencing, ‘omics sciences, and computational tools for analyzing big data. Pharmacogenomics is at the heart of this enterprise and holds the power to transform our understanding of human disease and response to treatment.

The authors performed a meta-analysis to find relationships between the transcriptional signatures of each SVZ population and the signatures resulting from exposure to small bioactive molecules. Out of the resulting catalog of molecules predicted to affect SVZ microdomain-specific lineages, Azim et al. [16] prioritized compounds for further study by the number of target genes and gene ontology (GO) pathway analysis. This led them to zero in on 2 compounds as having particularly noteworthy effects: AR-A014418, which appears to rejuvenate the NSC lineage, and LY-294002, which promotes development of oligodendrocytes by inhibiting PI3K/Akt signaling. The authors selected these as the most salient compounds/pathways to examine in mice. As hoped, the small molecules performed in vivo just as predicted by their pharmacogenomic analyses, promoting neurogenesis and oligodendrogenesis, respectively, in mice. Furthermore, the authors were able to promote regeneration in a mouse model of hypoxic brain injury, showing that GSK3β inhibitors allowed the recruitment of new oligodendrocytes and glutamatergic neurons into the cortex. This is obviously very relevant to hopes of future therapies for brain injury.

This study thus establishes the efficacy of pharmacogenetic approaches to generate a framework for further mechanistic and in vivo studies. Even more importantly, Azim et al. [16] have opened the door to a pharmacological approach to stimulating lineage-specific stem cell fate: until now, various groups have pursued genetic approaches or transplants of reprogrammed cells, which are difficult and invasive [21,22]. The results here show that promoting a particular lineage, such as oligodendrocytic or glutamatergic neuronal lineages, is a realistic goal. Lastly, it is worth noting that although the authors’ focus on early postnatal time points was necessary for technical reasons (to have enough cells to perform microarray analysis), the molecular discoveries they made point to this early postnatal period as a critical window for tissue repair because germinal matrix activity persists and the diversity of neural lineages produced is at its peak.

Future pharmacokinetic studies on the classes of proneurogenic or pro-oligodendrogenic compounds identified should be performed to determine their efficacy, safety, and required duration of treatment; long-term efficiency and effects in disease contexts also need to be determined, particularly in view of the multifactorial pathology that affects not only NSCs and their progeny but also the niches from which they originate and those that they eventually populate. Finally, while animal models of human disease provide a means of experimentally testing biological hypotheses, findings in human tissue or observations on live humans must validate the experimental findings. It is possible that microdomain-specific NSC populations are preserved in human brain organoids; these models could be the first step toward such validations. We should be cautious but hopeful. The work of Azim and collaborators [16] clearly brings us an important step closer toward specific agents to mobilize endogenous stem cells as a therapy for neurodegenerative and demyelinating disorders.

It used to be an article of faith that we could someday harness the potential of stem cells to stimulate regeneration of neural tissue. Now it is mostly a question of fate.

## Abbreviations

CMAP: Connectivity Map
GEM-TREND: Gene Expression data Mining Toward Relevant Network Discovery
GO: gene ontology
LINCS: Library of Integrated Network-based Cellular Signatures
NSC: neural stem cell
SGZ: subgranular zone
SPIED: Searchable Platform Independent Expression Database
SVZ: subventricular zone
TAP: transient amplifying cell
